# Gender diverse children deserve a good faith debate about gender-affirming care: Response to Cavve et al. (2024)

**DOI:** 10.1177/10398562251317335

**Published:** 2025-01-30

**Authors:** Andrew James Amos

**Affiliations:** 104397 Townsville, QLD

Dear Editor,

Ideally, the scientific method openly debates and rationally evaluates all viewpoints after considering all available evidence. By contrast, many gender-affirming care (GAC) advocates appear to believe that results and opinions that don’t support its rapid expansion must be suppressed by any means available.^1^

In my opinion, the letter by Cavve et al.^2^ responding to my article documenting the rapid increase in patients treated at Australia’s paediatric gender clinics^3^ (APGC) provides a good example of this strategy. They implicitly concede the main result and, rather than constructively engaging with my arguments or identifying errors in my analyses, accuse me of intentionally misrepresenting my research and the broader literature.

Others judging the good faith of the parties to the dispute should note that Cavve et al. don’t acknowledge that I provided Cavve with all my data and analyses and answered all his questions over multiple emails. Cavve did not seek clarification of any of the misrepresentations alleged in his letter, which he would have done if interested in good faith discussion. Neither do Cavve et al. report he refused the opportunity to discuss GAC in an online forum with myself and Hilary Cass, author of the most authoritative report on GAC currently available.^4^

Fortunately, *Australasian Psychiatry* readers do not have to accept Cavve et al.’s or my authority on who has indulged in misrepresentation. Indeed, cursory examination of their letter suggests that their refusal to debate critics is probably wise.

Most fundamentally, they argue I have misrepresented APGCs as ‘forbid[ding] clinicians to question or evaluate gender identity’.^2^ No such claim is made in my article – this is simply false.

Perhaps even more concerning is the claim that I misrepresented the ‘spectre that gender-affirming care may lead to neglect of existing mental health conditions’,^2^ while failing to acknowledge my explicit reference to evidence that GAC leads to this exact phenomenon, labelled ‘diagnostic overshadowing’ in the Cass Review.^4^

Cavve et al.’s treatment of detransition is also revealing. As they can’t deny that the APGCs’ clinical guidelines fail to mention detransition, they try to substitute statements about all gender identities being equally acceptable, and changing goals,^2^ neither of which actually represent detransition.

Finally, Cavve et al.^2^ refer to a different article by Cavve^5^ to imply that only 1% of APGC patients detransition and cite it as an example of ‘high-quality’ APGC research. This retrospective cohort study estimated detransition rates by imputing cause of discharge from APGC case notes, excluding 27 patients who reidentified with birth sex before or during assessment. The 1% estimate is based on two patients who reidentified with birth sex after initiation of puberty blockers or hormone therapy.

Apart from the methodological limitations of a retrospective cohort study based on case notes, the article arbitrarily focuses on medical transition, and it is unclear why the majority of patients who reidentified with birth sex were not considered to be detransitioning after social transition. In addition, it is impossible to accurately estimate lifetime detransition rates from status at discharge.

While I lack the space to address the other articles referenced by Cavve et al.,^2^ I encourage those interested in gender medicine to review a few at random to evaluate Cavve et al.’s claim that APGCs are producing ‘high-quality research’. This will quickly confirm the authoritative judgement of the Cass Review that ‘there continues to be a lack of high quality evidence’ regarding gender affirming care (p 20).^4^

I repeat the offer for a public debate regarding gender-affirming care with any or all of these authors.
